# Fine Mapping of Five Loci Associated with Low-Density Lipoprotein Cholesterol Detects Variants That Double the Explained Heritability

**DOI:** 10.1371/journal.pgen.1002198

**Published:** 2011-07-28

**Authors:** Serena Sanna, Bingshan Li, Antonella Mulas, Carlo Sidore, Hyun M. Kang, Anne U. Jackson, Maria Grazia Piras, Gianluca Usala, Giuseppe Maninchedda, Alessandro Sassu, Fabrizio Serra, Maria Antonietta Palmas, William H. Wood, Inger Njølstad, Markku Laakso, Kristian Hveem, Jaakko Tuomilehto, Timo A. Lakka, Rainer Rauramaa, Michael Boehnke, Francesco Cucca, Manuela Uda, David Schlessinger, Ramaiah Nagaraja, Gonçalo R. Abecasis

**Affiliations:** 1 Istituto di Ricerca Genetica e Biomedica, Consiglio Nazionale delle Ricerche (CNR), Monserrato, Italy; 2 Department of Biostatistics, Center for Statistical Genetics, University of Michigan, Ann Arbor, Michigan, United States of America; 3 Dipartimento di Scienze Biomediche, Università di Sassari, Sassari, Italy; 4 Shardna Life Sciences, Pula, Italy; 5 Gene Expression and Genomics Unit, Research Resources Branch, National Institute on Aging, Baltimore, Maryland, United States of America; 6 Department of Community Medicine, Faculty of Health Sciences, University of Tromso, Tromso, Norway; 7 Department of Medicine, University of Eastern Finland, Kuopio, Finland; 8 Department of Public Health, Faculty of Medicine, Norwegian University of Science and Technology, Trondheim, Norway; 9 Diabetes Prevention Unit, National Institute for Health and Welfare, Helsinki, Finland; 10 Hjelt Institute, Department of Public Health, University of Helsinki, Helsinki, Finland; 11 South Ostrobothnia Central Hospital, Seinajoki, Finland; 12 Institute of Biomedicine/Physiology, University of Eastern Finland, Kuopio, Finland; 13 Kuopio Research Institute of Exercise Medicine, Kuopio, Finland; 14 Laboratory of Genetics, National Institute on Aging, Baltimore, Maryland, United States of America; Georgia Institute of Technology, United States of America

## Abstract

Complex trait genome-wide association studies (GWAS) provide an efficient strategy for evaluating large numbers of common variants in large numbers of individuals and for identifying trait-associated variants. Nevertheless, GWAS often leave much of the trait heritability unexplained. We hypothesized that some of this unexplained heritability might be due to common and rare variants that reside in GWAS identified loci but lack appropriate proxies in modern genotyping arrays. To assess this hypothesis, we re-examined 7 genes (*APOE*, *APOC1*, *APOC2*, *SORT1*, *LDLR*, *APOB*, and *PCSK9*) in 5 loci associated with low-density lipoprotein cholesterol (LDL-C) in multiple GWAS. For each gene, we first catalogued genetic variation by re-sequencing 256 Sardinian individuals with extreme LDL-C values. Next, we genotyped variants identified by us and by the 1000 Genomes Project (totaling 3,277 SNPs) in 5,524 volunteers. We found that in one locus (*PCSK9*) the GWAS signal could be explained by a previously described low-frequency variant and that in three loci (*PCSK9*, *APOE*, and *LDLR*) there were additional variants independently associated with LDL-C, including a novel and rare *LDLR* variant that seems specific to Sardinians. Overall, this more detailed assessment of SNP variation in these loci increased estimates of the heritability of LDL-C accounted for by these genes from 3.1% to 6.5%. All association signals and the heritability estimates were successfully confirmed in a sample of ∼10,000 Finnish and Norwegian individuals. Our results thus suggest that focusing on variants accessible via GWAS can lead to clear underestimates of the trait heritability explained by a set of loci. Further, our results suggest that, as prelude to large-scale sequencing efforts, targeted re-sequencing efforts paired with large-scale genotyping will increase estimates of complex trait heritability explained by known loci.

## Author Summary

Despite the striking success of genome-wide association studies in identifying genetic loci associated with common complex traits and diseases, much of the heritable risk for these traits and diseases remains unexplained. A higher resolution investigation of the genome through sequencing studies is expected to clarify the sources of this missing heritability. As a preview of what we might learn in these more detailed assessments of genetic variation, we used sequencing to identify potentially interesting variants in seven genes associated with low-density lipoprotein cholesterol (LDL-C) in 256 Sardinian individuals with extreme LDL-C levels, followed by large scale genotyping in 5,524 individuals, to examine newly discovered and previously described variants. We found that a combination of common and rare variants in these loci contributes to variation in LDL-C levels, and also that the initial estimate of the heritability explained by these loci doubled. Importantly, our results include a Sardinian-specific rare variant, highlighting the need for sequencing studies in isolated populations. Our results provide insights about what extensive whole-genome sequencing efforts are likely to reveal for the understanding of the genetic architecture of complex traits.

## Introduction

In the past few years, genome-wide association studies (GWAS) have identified hundreds of genetic variants associated with quantitative traits and diseases, providing valuable information about their underlying mechanisms (for a recent example, see [1]). More than 2,000 common variants appear associated with over 200 conditions (as reported by the NHGRI GWAS catalog on 12/2010) and for a few, like age-related macular degeneration [2] and type 1 diabetes [3], these common variants already account for a large fraction of trait heritability. In contrast, for most complex traits and diseases, common variants identified by GWAS confer relatively small increments in risk and explain only a small proportion of trait heritability [4]. For example, for low-density lipoprotein cholesterol (LDL-C), GWAS based on up to ∼100,000 individuals examined at ∼2.5 million common variants [1], [5]–[6], have identified 35 loci associated with trait variation, with some also involved in modulating the risk of cardiovascular diseases. Common variants at these loci are estimated to account for 12.2% of the variability in LDL-C levels, about one-fourth of its genetic variance [1]. Several hypotheses have been formulated about the nature of the remaining heritability of lipid levels and other complex traits [4], [7], ranging from the potential role of copy number variants to contributions from a large number of common SNPs each with very small effects. In our view, common and rare variants that are poorly represented in common genotyping arrays might account for an important fraction of trait heritability. Ignoring these variants might not only preclude identification of important trait associated loci but also compromise estimates of heritability. Thus, fine mapping appears the logical next step after GWAS. Here, we have focused on seven genes located in five of the loci associated with LDL-C in our original GWAS for blood lipid levels (*APOE*, *APOC1*, *APOC2*, *SORT1*, *LDLR*, *APOB* and *PCSK9*) [5]. A sixth locus (corresponding to SNP rs16996148) that included a large number of genes and no obvious functional candidates was not further examined here. Together, the 5 SNPs identified in the original GWAS analyses of these 5 loci in >8,000 individuals (with follow-up genotyping of >10,000 individuals) explained only 3.1% of LDL-C variability. We set out to re-assess the contribution of these loci to trait heritability by evaluating a broader spectrum of variants. To catalog genetic variation in these regions, we first sequenced the exons and flanking regions of the seven genes in 256 unrelated Sardinians [8], each with extremely low or high LDL-C, and in an additional 120 HapMap samples (parents from the 30 CEU and 30 YRI trios). To assess the effect of identified polymorphisms, we genotyped detected variants and additional variants selected based on an early release of the 1000 Genomes Project in a cohort of 5,524 volunteers from the SardiNIA project [8]. Our results show that at these five loci, a combination of rare and common variants, some novel and some previously identified, are associated with LDL-C, and that, taken together they double the variance explained by the common variants detected in GWAS.

## Results

To refine the contribution of five loci implicated by GWAS in the variability of LDL-C, we sequenced the exons and flanking regions of seven genes in 256 unrelated Sardinians [8] with LDL-C levels that were either extremely low (116 individuals, mean LDL-C = 70.4±16.0 mg/dl) or high (140 individuals, mean LDL-C = 205.9±19.6 mg/dl) (Materials and Methods), as well as an additional 120 HapMap samples (parents from the 30 CEU and 30 YRI trios). Observed heterozygosity per base pair per individual was 1.28×10^−3^ in the selected Sardinian individuals, 1.31×10^−3^ in the CEU and 1.99×10^−3^ in the YRI.

Sequencing identified 782 variants, all submitted to dbSNP and now included in dbSNP releases 130 and later (for a complete list see Table S1). As expected, more variants were found in the HapMap YRI samples than in the HapMap CEU or in Sardinian individuals with extreme lipid levels (Table S2). Overall, we observed a 2∶1 trend for enrichment of rare variants (MAF<1%) in the high LDL-C group compared to the low LDL-C group, similar to the observation by Johansen and colleagues [9] (Table S3), but this enrichment was only statistically significant for *APOB* (*P* = 0.03 using an exact test). To test for LDL-C association, we used logistic regression to compare individuals in the two categories, yielding 10 variants (in *APOE*, *APOC1*, *SORT1*, *APOB*, and *PCKS9*) with *P*<0.1 (Table S4). Because of the modest number of sequenced individuals and because no signal reached significance after Bonferroni adjustment, we judged these initial association analyses – which focused only on sequenced samples and only at coding regions – inconclusive.

In addition to the loci discussed so far, our re-sequencing and genotyping effort also included *B3GALT4* and *B4GALT4*, two loci that approached genome-wide significance in our initial GWAS analysis (each with 5×10^−8^<p<5×10^−6^) [5]. SNPs in these loci did not reach genome-wide significance in two subsequent meta-analyses [1], [6] and were not significantly associated with LDL-C in the data generated here (Table 1 and Figure S1). Because we have no evidence that these two genes are associated with LDL-C, they are not discussed further. Variants identified in the two genes were also deposited in dbSNP.

**Table 1:** Association Analysis results.

Locus	SNPname	Type	Effect Allele/Other	Freq Effect Allele	Effect (SE)^a^	P-value	Genomic Annotation	Variance explained by the locus	Top GWAS SNP	Effect Allele/Other	Freq Effect Allele	Effect (SE)^a^	P-value	r2	Adjusted P-value	Variance explained by the locus
*PCSK9*	rs11591147	Metabochip	T/G	0.037	−0.380 (0.048)	2.90×10^−15^	**missense (R46L)**	1.19%	rs11206510	C/T	0.243	−0.106 (0.023)	5.71×10^−07^	0.101	0.013	0.23%
	rs2479415	1000G	C/T	0.413	0.076 (0.019)	7.50×10^−05^	8 Kb from *PCSK9*									
*SORT1*	rs583104	Metabochip	T/G	0.177	0.149 (0.024)	1.28×10^−09^	31 Kb from *SORT1* b	0.63%	rs599839	G/A	0.276	−0.148 (0.025)	1.43×10^−09^	0.991	0.90	0.61%
*B3GALT4*	rs28361085	1000G	C/T	0.073	0.114 (0.036)	0.00169	146 Kb from *B3GALT3*	0.22%	rs2254287	G/C	0.492	0.005 (0.018)	0.771	0.413	0.84	0.02%
*B4GALT4*	rs34507110	1000G	G/A	0.154	0.122 (0.030)	4.99×10^−05^	83 Kb from *B4GALT4*	0.48%	rs12695382	A/G	0.075	−0.074 (0.035)	0.035	0.795	0.48	0.03%
*APOB*	rs547235	1000G	A/G	0.187	−0.144 (0.024)	1.69×10^−09^	140 Kb from *APOB*	0.51%	rs562338	A/G	0.173	−0.139 (0.025)	1.43×10^−8^	0. 878	0.98	0.43%
*LDLR*	rs73015013	Metabochip	T/C	0.138	−0.155 (0.027)	1.12×10^−08^	9 kb from *LDLR*	1.17%	rs6511720	T/G	0.132	−0.160 (0.027)	1.71×10^−08^	0.934	0.97	0.59%
	rs72658864	Metabochip	C/T	0.005	0.626 (0.136)	3.90×10^−06^	**missense (V578A)**									
*APOC1/C2/E*	rs7412	Metabochip	T/C	0.037	−0.563 (0.048)	1.80×10^−31^	**missense (R176C) ** ***APOE***	3.33%	rs4420638^c^	G/A	0.097	0.218 (0.031)	4.67×10^−12^	0.0003	6.41×10^−10^	1.07%
	rs429358	Affy+Sanger	C/T	0.071	0.260 (0.036)	5.82×10^−11^	**missense (C130R) ** ***APOE***									

The left panel shows the association results at 7 loci. For each gene, the strongest variant is listed first, and any second detected independent signal is listed with results from the conditional analysis (Materials and Methods). The column Type indicates whether the SNP was directly genotyped (Metabochip) or imputed using 1000G reference haplotype (1000G) or the Sardinian reference panel (Affy+Sanger). The right panel shows the association results for the GWAS SNPs previously described [5], the correlation with the top SNP listed in the left panel, and its p-value in the conditional analysis (Adjusted P-value).

a Effect sizes are standardized (see Materials and Methods), and represent the change in trait LDL-C values associated with each copy of the reference allele, measured in standard deviation units.

b SNP rs583104 is also 1 Kb from *PSRC1* transcript.

c r^2^ = 0.967 with Metabochip second-independent SNP, rs429358. After adjusting for the two independent SNPs, rs7412 and rs429358, the p-value for rs4420638 was 0.5.

To increase the power to detect association, we genotyped 5,524 individuals in the SardiNIA cohort [8] using the Metabochip (see Materials and Methods). The chip included 285 variants newly discovered by sequencing, together with an additional 2,992 derived from an early analysis of 1000 Genome Project Pilot haplotypes (considering variants ±250 Kb from each gene). Of the 3,277 SNPs that were genotyped, 1,868 passed quality control filters (see Materials and Methods and Table S5). To further supplement the number of variants at each locus, we carried out two rounds of genotype imputation. First, we used haplotypes for 256 sequenced SardiNIA samples to impute genotypes for SNPs that failed assay design or genotyping on the Metabochip. Second, using the haplotypes of 60 CEU samples from the 1000 Genomes Pilot, we successfully imputed an additional 5,066 variants [10] (Materials and Methods and Table S5). After imputation, 7,488 SNPs were available for analysis, with an average minor allele frequency of 18% and an average imputation r^2^ of 0.84 for 5,620 imputed SNPs (554 and 5,066 from Sanger and 1000 Genomes imputations, respectively; see Table S5 for gene specific counts).

**Figure 1:**
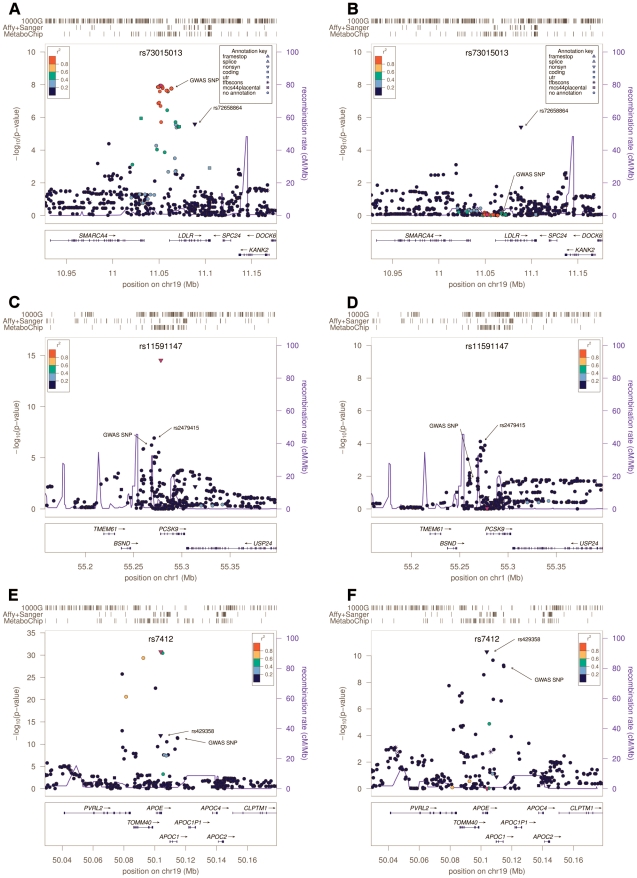
Regional Association plots. Association results around *LDLR*, *PCSK9* cluster and *APOE*. In each panel, the box at left (A, C and E) shows the association results in the main analysis; and at right (B, D and F) the results after conditioning for the strongest associated variant, highlighted with a purple dot in both plots, and its name written at the top. Arrows highlight independent signals and the most associated SNP detected in the previous GWAS [5]. Each SNP is also colored according to its LD (r^2^) in Sardinians with the top variant, with symbols that reflect genomic annotation as indicated in the legend. The rugs above indicate the position of the SNPs that were analyzed by direct typing (MetaboChip), or imputed by using haplotypes from sequenced samples (Affy+Sanger) or 1000 Genomes haplotypes (1000G). Plots were drawn using the LocusZoom standalone version [37]. Genomic coordinates are given according to build 36 (hg18).

At three loci, *SORT1*, *APOB* and *LDLR*, GWAS-identified variants were very strong proxies for the best available association signal, with similar allele frequencies and r^2^>0.88 (Table 1, Figure 1A and Figure S2). In those three genes, the variant showing strongest association was non-coding and not in strong linkage disequilibrium (r^2^>0.4) with any tested coding variant. The most strongly associated marker at the *SORT1* locus, rs583104 (p-value = 1.2×10^−9^) was in high LD (r^2^ = 0.77) with rs12740374 (p-value = 2.2×10^−8^), an intronic SNP in the *CELSR2* gene that alters the hepatic expression of the *SORT1* gene by creating a C/EBP (CCAAT/enhancer binding protein) transcription factor binding site [11]. Both markers were genotyped, so that under the hypothesis that rs12740374 is the causal variant underlying this association signal, the modest difference in p-values may be attributable to statistical fluctuation.

At the remaining two loci, *APOE* and *PCSK9*, evidence for association peaked at low frequency (1–5%) variants not in strong linkage disequilibrium with the original GWAS signals. In both cases our analyses pointed to variants that were well studied in other contexts, but which are not included in typical GWAS panels or in the HapMap panel of European haplotypes commonly used to impute missing genotypes. Thus these variants were missed in previous GWAS analyses. In *PCSK9*, variant rs11591147, which leads to a non-synonymous R46L change in exon 1, was more strongly associated (*P* = 2.9×10^−15^, frequency (T) = 0.037, effect = −12.9 mg/dl; Table 1) than GWAS variant rs11206510, a SNP ∼10 Kb upstream of the transcription start site of the gene (*P* = 5.7×10^−7^, frequency (C) = 0.24, effect = −3.7 mg/dl) (Figure 1C). Furthermore, rs11591147 explained the GWAS association signal (association at GWAS variant rs11206510 became non-significant (*P* = 0.013) when non-synonymous variant R46L/rs11591147 was included as a covariate, Figure 1D). This coding variant has been previously implicated in the regulation of blood lipid levels, including LDL-C, and in the susceptibility to coronary and ischemic heart disease [12]–[13]. At the *APOE* gene cluster, the strongest evidence of association was observed at the missense variant (R176C, also known as R158C [14]) rs7412 (*P* = 1.8×10^−31^, frequency (T) = 0.037, effect = −18.8 mg/dl) (Figure 1E). This variant did not account for the previously reported GWAS signal; marker rs4420638 indeed remained significantly associated (*P* = 6.4×10^−10^) after adjusting for rs7412. The missense variants at *APOE* and *PCSK9* were not typed in the HapMap II data set, and were only recently added to genotyping arrays (Illumina 1MDuo). Thus they have not been assessed by any GWAS reported to date.

We next conditioned on the top association signal at each of the 5 loci and sought to identify additional independently associated variants. To declare statistical significance at secondary signals, we used a p-value threshold of 1×10^−4^; corresponding to an adjustment for 500 independent tests across the five regions examined. At *LDLR*, we found an independently associated rare missense variant (rs72658864/V578A, *P* = 2.5×10^−6^ in the basic model, *P* = 3.9×10^−6^ in the conditional model, frequency (C) = 0.005; effect = 23.7 mg/dl) (Table 1 and Figure 1B). This variant appears to be specific to Sardinia (where we identified it in our SardiNIA cohort [8] by Sanger sequencing in 3 out of 256 individuals with extreme LDL-C; by Illumina genotyping in 51 out of 5,800 randomly ascertained individuals; and by Solexa sequencing in 1 out of 505 individuals, unpublished data). It is absent in the HapMap data set, not detected in 280 Northern European individuals sequenced within the 1000 Genomes Project, and monomorphic in >10,000 Finnish [15]–[16] and Norwegian [17]–[19] individuals genotyped with the MetaboChip (Materials and Methods, Table S6 and Table S7). Reassuringly, the variant was also observed, albeit with a lower frequency (0.00035), in TaqMan genotyping an independent sample of 5,661 Sardinians from different villages in Sardinia [20] (Materials and Methods). The change in lipid levels associated with this rare variant (23.7 mg/dl) is 4 times greater than that observed for the strongest associated common variant at the locus (5.7 mg/dl for rs73015013). At the *APOE* locus, we found a strong independent signal at non-synonymous variant rs429358 (C130R, also known as C112R [14]) (Table 1 and Figure 1F)(*P* = 1.2×10^−12^ in the basic model, *P* = 5.8×10^−11^ in the conditional analysis, frequency (C) = 0.071, effect = 9.3 mg/dl), which, together with rs7412, defines the three major isoforms of *APOE* (ε2, ε3 and ε4) [14], [21]. This variant strongly correlates (r^2^ = 0.96) with the originally reported GWAS signal, rs4420638 (*P* = 4.6×10^−12^, frequency (G) = 0.097, effect = 7.8 mg/dl). So, at this locus, the initial GWAS analysis picked up one independent signal (a proxy of rs429358/C130R) but missed the strongest associated variant in the region (rs7412/R176C). There was no clear evidence for residual association after accounting for the two missense variants (Figure S3). Interestingly, the frequency of the derived allele C at rs429358 was remarkably lower in Sardinia (freq = 0.07, see Table 1) than that observed in the Finnish and Norwegian individuals (see Table S7) and several other European ancestry samples (freq∼0.20) [22]–[24], resulting in a strikingly lower frequency of the ε4 haplotype (2.5% vs. 15%) [22]. Finally, at *PCSK9*, we observed a possible independent association at SNP rs2479415, in the non-coding region flanking the transcript (*P* = 1.1×10^−7^ in the basic model, *P* = 8×10^−5^ in the conditional model, frequency (T) = 0.59, effect = −3.6 mg/dl) (Table 1 and Figure 1D). This variant showed an independent trend also in ∼10,000 Finnish and Norwegian individuals (one-sided *P* = 0.055 after conditioning for rs11591147).

When the 5 GWAS SNPs were replaced by the 8 variants described here (1 each for *SORT1* and *APOB*, 2 for *APOE*, *PCSK9* and *LDLR*) the variance accounted for by those loci increased from 3.1% to 6.5%. Similar estimates were also obtained with ∼10,000 Finnish and Norwegian individuals, where, on average, analysis of these 8 variants increased variance explained from 3.5% to 7.1% (Table 2 and Materials and Methods).

**Table 2:** Heritability estimates in all study samples.

Study	N samples	Variance explained by 5 GWAS SNPs	Variance explained by 8 SNPs
SardiNIA	5,382	3.1%	6.5%
Norwegian T2D	1,171	5.8%	9.3%
Norwegian controls	1,436	3.1%	8.5%
Finnish T2D	1,742	2.1%	5.0%
Finnish controls	5,678	3.4%	7.0%
*Average Finnish and Norwegian*	*10,027*	*3.5%*	*7.1%*

The table shows the LDL-C variance accounted for by the 5 GWAS SNPs and the 8 SNPs here described in all studies. A sample size weighted average estimate is given for the Finnish and Norwegian samples.

## Discussion

We conducted fine mapping of five loci associated with LDL-C at an unprecedented level of resolution. In particular, we sequenced individuals with extreme phenotype levels, and subsequently genotyped variants identified by us and by the 1000 Genomes Project in a larger sample. In a final step we also imputed additional variants in the region to account for limitations of genotyping assay design. At all but one of the loci, *APOB*, the most strongly associated variant was directly genotyped or sequenced, suggesting that our initial selection included the crucial variants. In three loci, we found strongly associated rare or low frequency variants – which (except for a variant in LDLR, which appears to be specific to Sardinia) had been extensively characterized in previous non-GWAS studies. In these cases, although the associated variants had been previously described, they had not been thoroughly examined in together with GWAS associated variants at the same loci – so that the relative contributions of GWAS identified SNPs and previously described variants remained unclear.

In summary, we observed that:

- At *SORT1* and *APOB* loci, association peaked at variants with similar effect size and frequency to the variants identified in GWAS;
- At the *LDLR* locus, in addition to confirming the GWAS signal, a rare variant with a large effect was found. This variant is currently unique to the island of Sardinia;
- At the *APOE* locus, an independently associated low frequency variant was identified. The signal was previously missed in GWAS because the variant was not included in the available genotyping chips or in the HapMap reference panels. An independently associated common variant similar in frequency and effect size to the original GWAS signal was also identified.
- At the last locus, *PCSK9*, the GWAS signal could be explained by a low frequency coding variant not included in the available GWAS genotyping chips or in the HapMap reference panels. Furthermore, there was evidence for one other independently associated variant.

The strongest signals identified at *APOE* (both variants) and *PCSK9* (the top hit) are likely to be the causal variants underlying the association signals. For *SORT1*, the variant exhibiting strongest association appears to be in strong linkage disequilibrium with a recently proposed functional polymorphism. In contrast, biological interpretation remains unclear for the other identified polymorphisms and requires further studies. Our results lead to several important major conclusions. First, it is striking that prior LDL-C GWAS have often missed signals due to low frequency variants (in two of the loci examined here, we identified strongly associated variants with frequency 1–5% that were missed in the original GWAS, because they were untyped or missing on imputation panels and poorly tagged by nearby SNPs). Sequencing in individuals with extreme trait values, along with large-scale imputation and genotyping, provided a better evaluation of the contribution of these loci to variation in LDL-C levels. A similar design was recently used to fine-map loci associated with fetal hemoglobin levels, a trait for which three loci can now account for about half of total variance [25].

Second, we show that in one of the five loci we fine-mapped, a previously missed low frequency variant can account for the GWAS signal – consistent with the hypothesis that at least some GWAS signals will be due to disequilibrium with nearby low frequency or rare variants [26]. There is considerable debate on how frequently this scenario will occur [27]. Our observations are compatible with some of the arguments made on both sides of this debate [26]–[27]. For example, in the case of *PCSK9*, a single low frequency variant explains the observed common variant association signal but did not appear to reduce the ability of the genome-wide association study to localize the functional element of interest. Furthermore, the effect of this variant was too small to be detectable in most linkage studies (including our own linkage analysis of >35,000 relative pairs in Sardinia). Further, a single low frequency variant (and not a cluster of variants) was sufficient to explain this association signal.

Finally, our results show that if estimates are based only on the common variation assessed through GWAS, heritability at identified loci is likely to be underestimated. A more complete dissection, including common, low frequency and rare variants (some of which will be population specific), dramatically increased the proportion of heritability associated with the 5 loci examined here, from 3.1% to 6.5%. Notably, the variance explained by each locus increased when a rare variant was found as a primary or secondary hit (*LDLR*, *APOE* and *PCSK9*), even when the top GWAS SNP highly correlates with a strong association signal (*LDLR* and *APOE*). By contrast, only slight improvements were observed at loci where the most associated marker highly correlated with the GWAS SNPs and there was no evidence for additional independent signals, even when the GWAS variant is unlikely to be functional (*SORT1* and *APOB*).

Genome-wide association studies have proven to be an extremely productive strategy for identifying regions of the genome associated with complex traits, often leading to unexpected insights into complex trait biology. A major efficiency of these studies is that, by focusing on a subset of variants that can be genotyped using array based platforms, they can conveniently and economically survey many common variants in large numbers of individuals. Our results emphasize the utility of these genome-wide studies in identifying trait association regions, but also emphasize that caution is needed when genome-wide study results are used to quantify the overall contribution of a locus to trait heritability. In our opinion, and consistent with our results, accurate estimates of heritability will require more extensive examination of each identified locus.

Broadly, this observation is consistent with recent simulation studies [28] which explore, in the context of a dichotomous trait, the relationship between effect sizes observed at GWAS SNPs and at true causal variants for the same locus. These simulation studies suggest that, most of the time, effect sizes estimated from GWAS would be similar to true effect sizes but that, some of the time, effect sizes estimated from GWAS might substantially underestimate the true effect size – especially in a scenario where rare variants are more likely to be causal. In cases where the effect size was underestimated by GWAS variants, a noticeable increase in heritability ensues.

It is also interesting to note that the effect sizes estimated here for rare and low frequency variants (all >10 mg/dl) are larger than the effect sizes of any of the common variants identified in GWAS studies. Effect sizes of more rare alleles associated with familial hypercholesterolemia are even larger (see [29] for examples of *PCSK9* variants with effects >100 mg/dl). This is consistent with the intuition that alleles with a large impact on LDL-C levels will be under strong natural selection and will, thus, be prevented from reaching high frequency in the population. Although rare and low frequency alleles with more modest impacts on LDL-C values are also likely to exist, we cannot detect them using available sample sizes and their detection must await studies of much larger sample sizes.

In conclusion, these results underline that the subsequent sequencing of the coding regions around GWAS associations in individuals with extreme values followed by large scale imputation and genotyping is an important step in assessing the contribution of associated genomic regions to trait heritability. If similar trends to those described here are observed at the remaining LDL-C associated loci, extending our approach described to all known LDL-C susceptibility loci could lead to an increase in the proportion of variance they explain from ∼12% to ∼24%, exceeding half of the genetic variance for this trait. Due to economic considerations, our sequencing efforts focused on the coding regions of each gene and only on genes that appeared very likely to be involved in lipid metabolism. In each locus, we augmented the set of discovered variants with variants discovered by the 1000 Genomes Project, but that will likely miss very rare as well as population specific variants. We expect that more extensive fine-mapping efforts that more comprehensively examine non-coding regions could identify additional trait associated variants. Ultimately, unbiased whole genome sequencing based association analyses might be required to fully explain the heritability of a trait like LDL-C, facilitating the comprehensive assessment of rare, population specific, and non-SNP variation. In the meantime, directed sequencing and large scale genotyping appears to be a promising approach.

## Materials and Methods

### Ethics statement

All individuals studied and all analyses on their samples were done according to the Declaration of Helsinki and were approved by the local medical ethics and institutional review committees.

### Samples description

The SardiNIA project is a population based study of aging-related traits that includes 6,148 related individuals from the Ogliastra region of Sardinia, Italy [8], [30]. During physical examination, a blood sample was collected from each individual and divided into two aliquots, one for DNA extraction and the other to characterize several blood phenotypes, including lipids levels. Specifically, LDL-C values were derived using the Friedwald formula that combines HDL and total cholesterol levels. The Finnish and Norwegian individuals are Type 2 Diabetes patients and unaffected individuals collected from several studies. Specifically, Finnish studies are: Dehko 2D 2007 (D2D 2007), Dose Responses to Exercise Training (DrsEXTRA), Diabetes Prevention Study (DPS), FUSION stage 2 [15] samples (from ACTION LADA, D2D 2004, FINRISK 1987, FINRISK 2002, Health 2000, Savitaipale) and Metabolic Syndrome in Men (METSIM) [16]; Norwegian studies are: The Nord- Trøndelag Health Study (HUNT 2) [17]–[18] and The Tromsø Study (TROMSØ) [19]. Baseline clinic characteristics of the SardiNIA, Finnish and Norwegian studies are reported in Table S7.

The independent Sardinian sample used for assessing the frequency of the rare variant at *LDLR* consists of 5,661 individuals belonging to 884 families enrolled in the SharDNA study [20], which recruited volunteers from a cluster of villages located in the Ogliastra region: Talana, Urzulei, Baunei, Triei, Seui, Seulo, Ussassai, Perdasdefogu, Escalaplano and Loceri. Observed heterozygotes were unrelated to those observed in the SardiNIA study based on demographic records to track origin of individuals up to 10 generations.

### Sequencing

Sequencing of the 256 Sardinians and the 120 HapMap samples (parents from the 30 CEU and 30 YRI trios) was carried out at the University of Washington Genome Sequencing Center through the NHLBI Resequencing & Genotyping Service (Debbie Nickerson, PI). To select the 256 individuals to be sequenced, we adjusted LDL levels by age and sex and then identified individuals in the top and bottom 5% of the distribution (individuals under lipid-lowering therapy were not considered). Among those, we selected all unrelated individuals who had at least one sibling in the study and were genotyped with 500 K or 10 K arrays [30], to facilitate downstream follow-up and imputation analyses.

Among the 782 variants detected by sequencing, two loss-of-function variants were observed. However, these were identified only on HapMap samples (see Table S8). A common in-frame insertion in *APOB* was observed in Sardinia and in HapMap CEU samples but was not associated with LDL-C after multiple testing adjustment (rs17240441, *P* = 3.0×10^−4^; see Figure S1C and S1D, Table S8). The observed heterozygosity per bp/per individual was 0.00128, 0.00131 and 0.00199 in Sardinia, CEU and YRI samples, respectively. Concordance rate of HapMap II and III phases genotypes with those obtained from Sanger sequencing was 99.63%, while a lower rate (98.1%) was observed with genotypes obtained from the low-pass sequencing 1000 Genomes Project (43 CEU and 42 YRI samples were common between the two datasets), indicating the slightly lower accuracy of next-generation sequencing technologies and in particular of low-pass sequencing approaches [31].

### Genotyping

Genotyping was carried out with Metabochip arrays (Illumina), which were designed in collaboration with several international consortia [5], [32]–[33] with the aim to fine map association loci detected through GWAS for a variety of traits. Part of the design included a set of wild-card SNPs chosen by individual research groups, and the SardiNIA study promoted the inclusion of all variants detected by sequencing individuals with extreme LDL-C values. In particular, assays were successfully designed for 285 of the 782 variants discovered by sequencing and 178 passed quality controls filters (some of those were polymorphic only in HapMap individuals, but we included all detected variants on the chip to assess heterozygosity on a large sample). Briefly, 3,277 variants were included on MetaboChip, and 1,868 passed quality checks. For a detailed description of markers discarded by each filter see Table S9. Concordance rate of Sanger and Metabochip genotypes was 99.47% at QCed markers, evaluated comparing genotypes of the 256 sequenced samples.

Metabochip genotyping was performed using Illumina Infinium HD Assay protocol with Multisample Beadchip format, and GenomeStudio was used for genotype calling. All samples had a call rate>98%, and there was no evidence for mis-specified family relationships (evaluated using Relpair software [34]). We discarded markers if any of the following was true: a) call rate <95%, b) MAF = 0, c) Hardy-Weinberg Equilibrium P<10^−6^ or d) excess of Mendelian Errors (Table S9).

A total of 5,524 Sardinian individuals were genotyped, of which 5,382 had lipid measurements available and were not under lipid lowering therapy. In the Finnish and Norwegian studies, a total of 10,823 samples were genotyped, of which 10,027 had LDL-C measurement available and were not under lipid lowering therapy.

Genotyping of the rare *LDLR* variant rs72658864 on the SharDNA samples was carried out using TaqMan single SNP genotyping assays (Applied Biosystems). Given the rarity of the variant, DNA of a known heterozygote from the SardiNIA project was included in each well plate to allow detection of intensities of both alleles. The genotype of this sample was called as heterozygote in all plates.

### Imputation and statistical analyses

To better represent genomic variation, we merged genotypes from the 256 sequenced Sardinian samples with genotypes available from Affymetrix 500 K [30] and/or Metabochip for all variants +/−2 Mb spanning the gene's transcript. We then phased the haplotypes using MACH [10] and used this reference set of haplotypes to impute sequence variants in the rest of the cohort [35]. We then focused on variants within +/250 Kb of the gene transcript. To further fine map the region, we used 120 haplotypes from the 60 CEU samples sequenced within the 1000 Genomes Project (June 2010 release of haplotypes based on March 2010 genotypes release) to impute variants outside the coding regions and flanking sequences targeted in our sequencing study. MACH software was used for imputation, with the same sized window used for the Sardinian-based imputation (+/−2 Mb). The results obtained with these two rounds of imputation are identified in the text, as well in table and figure legends, as “Affy+Sanger” and “1000G”, respectively.

For association, LDL-C levels were adjusted for age, age squared and sex, and the distribution of residuals was normalized using a quantile transformation. The association test was performed using Merlin (–fastassoc option), which uses a variance component framework to account for genetic correlation across family members [35]–[36].

Comparison of imputed genotypes with experimental genotypes, carried out on a set of 1,097 individuals that were genotyped with the 6.0 Affymetrix Arrays (unpublished data), showed that the average per genotype error rate between imputed and experimental genotypes was 3.7% and 4.1% for imputations based on 1000 Genomes and Sanger haplotypes, respectively.

In the Finnish and Norwegian studies we applied a similar strategy to analyze variants (rs547235 and rs562338 on *APOB*, rs2479415 on *PCSK9* and rs429358 on *APOE*) that were not included on Metabochip. We defined a set of reference haplotypes of the 60 HapMap CEU founders by merging genotypes from the 1000 Genomes project and those from our Sanger sequencing, using SNPs located +/−2 Mb of *APOB*, *PCSK9* and *APOE*. We then used this reference panel to carry out imputation and successively used imputed dosages for testing association with LDL-C. Association analysis was performed using the same trait transformation and covariates as in the SardiNIA study. Imputation and association tests were performed separately for Finnish diabetics (N = 1,742), Finnish non-diabetics (N = 5,678), Norwegian diabetics (N = 1,171) and Norwegian non-diabetics (N = 1,436). Results were then meta-analyzed using an inverse-variance method, which combines p-values from each study using weights proportional to the variance of the beta coefficient (effect) (Table S7). A combined estimate of allele frequencies was obtained using the same weights.

### Variance explained

We evaluated the variance explained by a set of markers by including all of them into the linear model in addition to the clinical covariates (age, age squared, gender), and by subtracting the variance explained by this model versus the basic model (only clinical covariates). Analyses were performed using the lmekin function in R kinship package which uses a variance component framework to account for genetic correlation across family members. In particular, since variance is not purely additive across loci, heritability in Table 2 has been calculated using all 8 SNPs (or 5 SNPs) in the model rather than adding values observed at specific loci (Table 1). For the Finnish and Norwegian samples, the LDL-C variance explained was calculated in each study group separately, and a combined estimate was calculated by weighting each study according to its sample size (Table 2).

### Conditional analyses

We conducted conditional analyses to test for residual associations after accounting for a key SNP. The procedure consists of adding a SNP into the regression model as covariate and testing the effect of another SNP. Specifically, we performed this analysis by adding the strongest associated variant (key SNP) as covariate in order to test 1) whether that variant could explain the GWAS association signal; and 2) if additional independent signals were present. For the latter analysis, a threshold of P<1×10^−4^ was used to declare significance, corresponding to a Bonferroni threshold for 500 independent tests. A graphical representation of association results from the conditional analysis is shown in Figure 1B, 1D, 1F and in Figure S2B and Figure S2D.

### URLs

MACH software: http://www.sph.umich.edu/csg/abecasis/mach/;

HapMap project: http://www.hapmap.org/;

1000 Genomes Project: http://www.1000genomes.org/;

1000 Genomes Haplotypes for imputation:

http://www.sph.umich.edu/csg/abecasis/MACH/download/1000G-2010-06.html;

Locus Zoom: http://csg.sph.umich.edu/locuszoom/

R kinship package http://cran.r-project.org/web/packages/kinship/index.html

## Supporting Information

Figure S1 Association results at *B4GALT4(A)* and *B3GALT4 (B)*. Similarly to Figure 1, the strongest associated variant is highlighted with a purple dot, and its name written nearby. Arrows highlight independent signals and the most associated SNP detected in our original GWAS. Each SNP is also colored according with its LD (r^2^) in Sardinians with the top variants, with symbols that reflect genomic annotation, as in Figure 1. The rugs on top indicate the position of the SNPs that were analyzed by direct typing (MetaboChip), imputed by using haplotypes from sequenced samples (Affy+Sanger) or imputed by using 1000 Genomes haplotypes (1000G).
(TIF)

Figure S2 Association results at *SORT1* and *APOB*. Similar to Figure 1, the box at left (A and C) shows the association results in the main analysis; and at right (B and D) show the results after conditioning for the strongest associated variant, highlighted with a purple dot in both plots, and its name written at the top. Arrows highlight the independent signal (if any) and the most associated SNP detected in our original GWAS. Each SNP is also colored according with its LD (r^2^) in Sardinians with the top variant (used as covariate), with symbols that reflect genomic annotation, as indicated in the legend in panel A. The rugs on top indicate the position of the SNPs that were analyzed by direct typing (MetaboChip), imputed by using haplotypes from sequenced samples (Affy+Sanger) or imputed by using 1000 Genomes haplotypes (1000G).
(TIF)

Figure S3 Association results at *APOE* after adjusting for the two missense variants. The figure shows the association results at *APOE* after adjusting for the two independent signals rs7412 (indicated with a purple dot) and rs429358 indicated with an arrow. Each SNP is also colored according with its LD (r^2^) in Sardinians with the top variant (rs7412) in the main analysis, with symbols that reflect genomic annotation, as indicated in the legend. The rugs on top indicate the position of the SNPs that were analyzed by direct typing (MetaboChip), imputed by using haplotypes from sequenced samples (Affy+Sanger) or imputed by using 1000 Genomes haplotypes (1000G).
(TIF)

Table S1 List of all variants detected by sequencing in the 256 Sardinians and 120 HapMap Samples and relative counts (given on a separate excel file). The table lists the variants detected by Sanger sequencing, their genomic position in build 36, the corresponding alleles, the biological function and the observed frequency in Sardinians, CEU and YRI samples.
(XLS)

Table S2 Summary of variants detected by sequencing in the 256 Sardinians and 120 HapMap Samples. The table summarizes the variants detected by sequencing in different types of biological function.
(DOCX)

Table S3 Enrichment of rare variants (MAF<0.01). The table lists the number of carriers of coding mutations (MAF<0.01) for each gene in individuals with high or low LDL-C levels. Shaded rows indicate whether a trend for enrichment is observed, although significance was clear only at *APOB*.
(DOCX)

Table S4 Case-control association analysis results. Association signals showing a p-value<0.1 when comparing individuals with high and low LDL values.
(DOCX)

Table S5 Statistics of detected genotyped and imputed SNPs for each region (+/−250 Kb from gene's transcript). The table summarizes the variants detected and analyzed in each step (sequencing, genotyping, imputation) for each gene.
(DOCX)

Table S6 Clinical characteristics of study cohorts. The table describes the clinical characteristics for the Sardinians, Finnish and Norwegian populations used for association analyses.
(DOCX)

Table S7 Association results in the Finnish and Norwegian individuals. The table describes the association results for all SNPs in the diabetics and non-diabetics Finnish and Norwegian samples.
(DOCX)

Table S8 Loss-of-function and in-frame insertion variants identified by sequencing and their respective frequencies on each population. The table reports the specific loss-of-function and in-frame insertion variants detected in *APOB* and *PCSK9* genes, their genomic position in build36 and frequencies in the 256 Sardinians and 120 CEU and YRI populations.
(DOCX)

Table S9 Metabochip Genotype Quality Control Details. Statistics of quality controls filters. Note that a marker could have failed more than one check.
(DOCX)
