# Supplementary figures and images for: Fine Mapping of Five Loci Associated with Low-Density Lipoprotein Cholesterol Detects Variants That Double the Explained Heritability
Source: PLoS Genet. 2011 Jul 28;7(7):e1002198. doi: 10.1371/journal.pgen.1002198 (PMC3145627; doi:10.1371/journal.pgen.1002198)

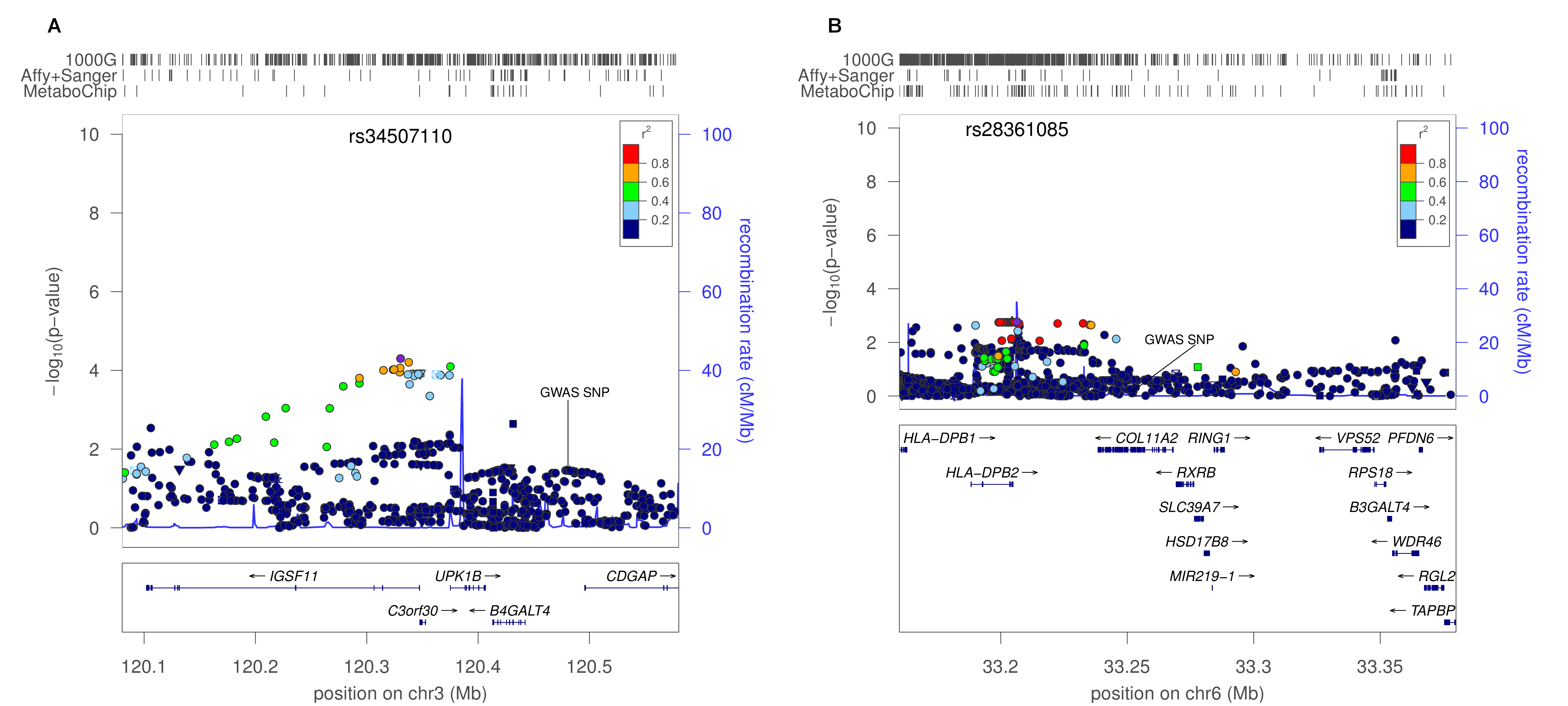

Supplement: Figure S1 — Association results at B4GALT4(A) and B3GALT4 (B). Similarly to Figure 1, the strongest associated variant is highlighted with a purple dot, and its name written nearby. Arrows highlight independent signals and the most associated SNP detected in our original GWAS. Each SNP is also colored according with its LD (r2) in Sardinians with the top variants, with symbols that reflect genomic annotation, as in Figure 1. The rugs on top indicate the position of the SNPs that were analyzed by direct typing (MetaboChip), imputed by using haplotypes from sequenced samples (Affy+Sanger) or imputed by using 1000 Genomes haplotypes (1000G). (TIF) [file pgen.1002198.s001.tif]

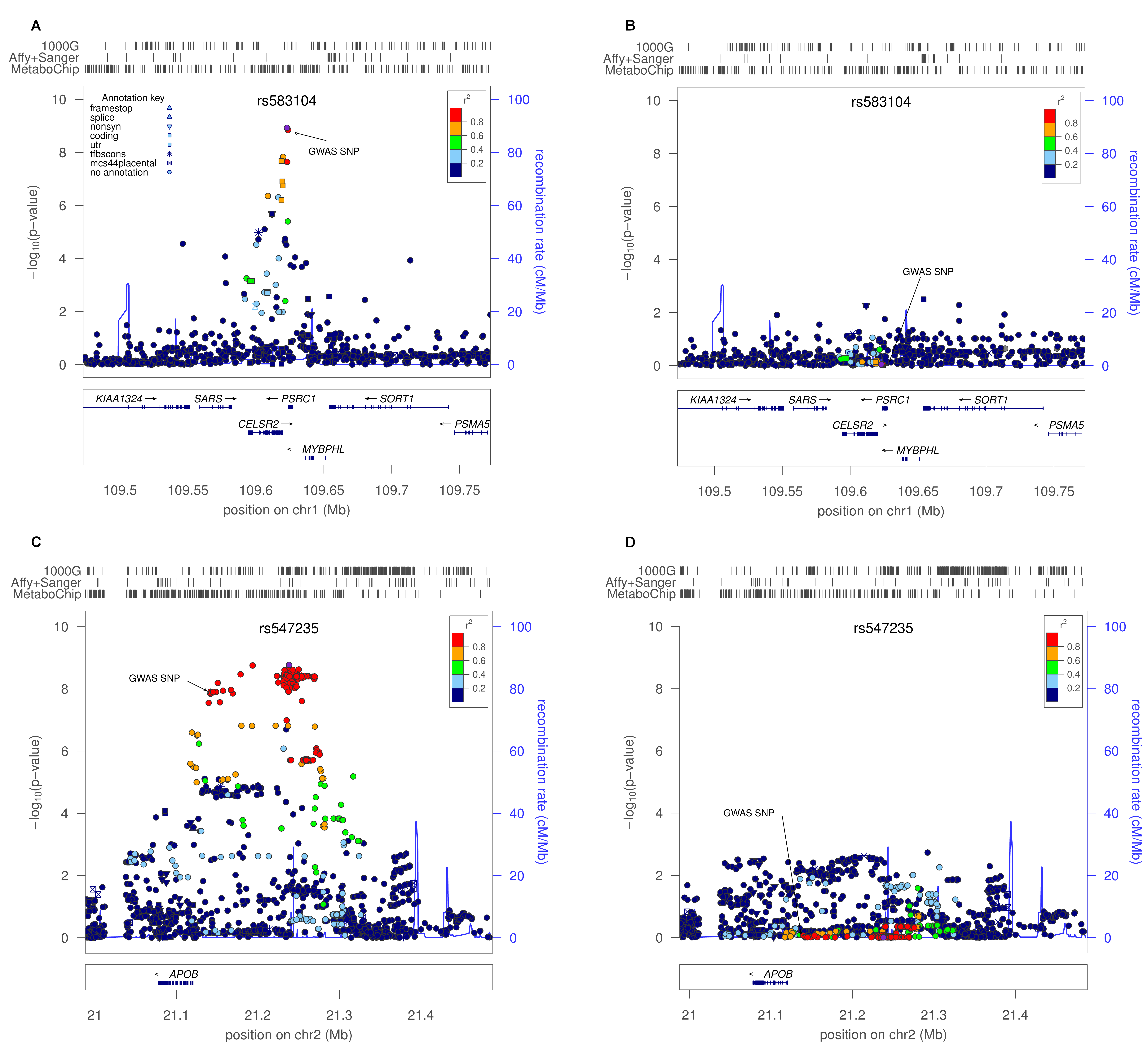

Supplement: Figure S2 — Association results at SORT1 and APOB. Similar to Figure 1, the box at left (A and C) shows the association results in the main analysis; and at right (B and D) show the results after conditioning for the strongest associated variant, highlighted with a purple dot in both plots, and its name written at the top. Arrows highlight the independent signal (if any) and the most associated SNP detected in our original GWAS. Each SNP is also colored according with its LD (r2) in Sardinians with the top variant (used as covariate), with symbols that reflect genomic annotation, as indicated in the legend in panel A. The rugs on top indicate the position of the SNPs that were analyzed by direct typing (MetaboChip), imputed by using haplotypes from sequenced samples (Affy+Sanger) or imputed by using 1000 Genomes haplotypes (1000G). (TIF) [file pgen.1002198.s002.tif]

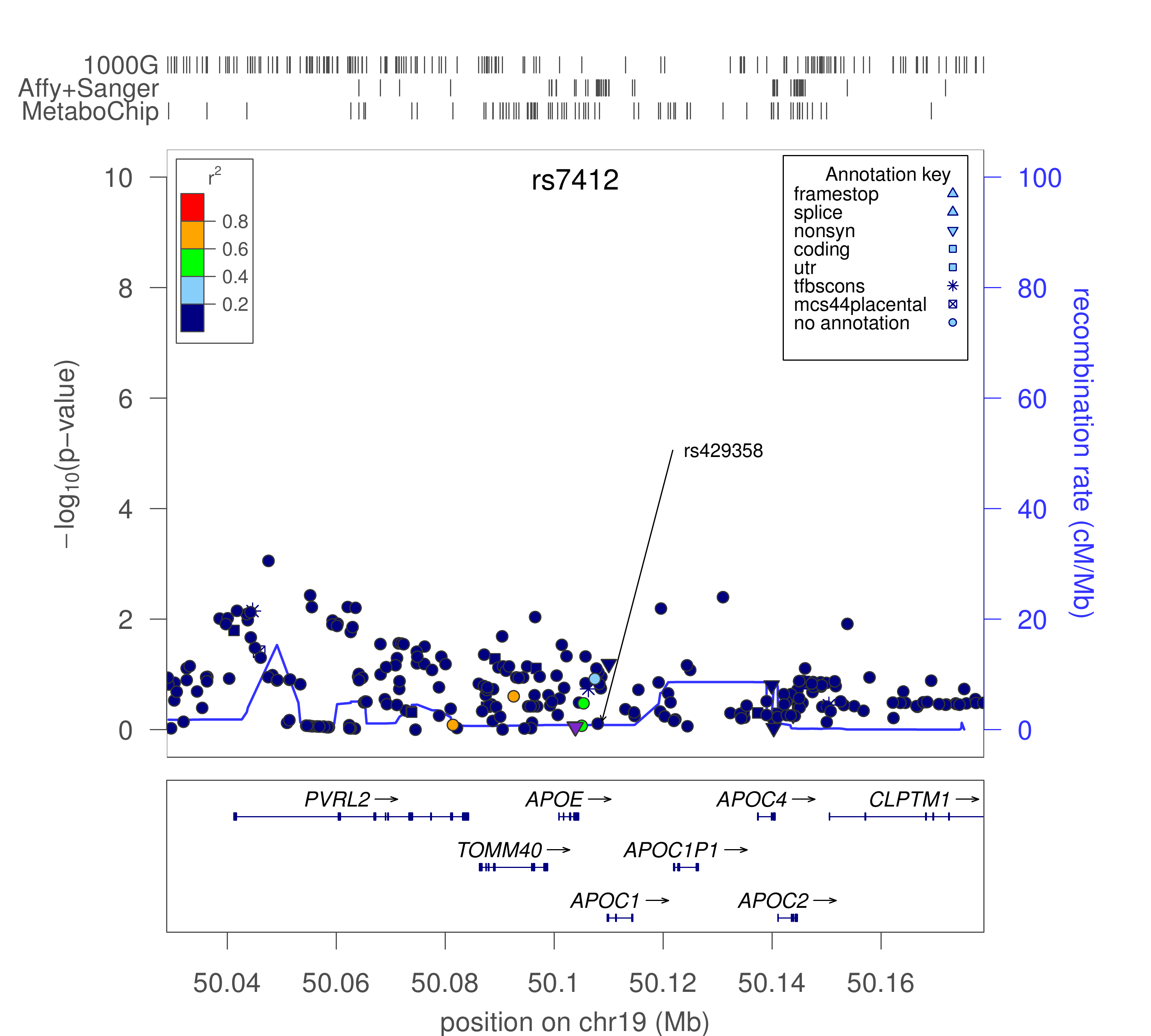

Supplement: Figure S3 — Association results at APOE after adjusting for the two missense variants. The figure shows the association results at APOE after adjusting for the two independent signals rs7412 (indicated with a purple dot) and rs429358 indicated with an arrow. Each SNP is also colored according with its LD (r2) in Sardinians with the top variant (rs7412) in the main analysis, with symbols that reflect genomic annotation, as indicated in the legend. The rugs on top indicate the position of the SNPs that were analyzed by direct typing (MetaboChip), imputed by using haplotypes from sequenced samples (Affy+Sanger) or imputed by using 1000 Genomes haplotypes (1000G). (TIF) [file pgen.1002198.s003.tif]
