# Supplementary material for: Fine Mapping of Five Loci Associated with Low-Density Lipoprotein Cholesterol Detects Variants That Double the Explained Heritability
Source: PLoS Genet. 2011 Jul 28;7(7):e1002198. doi: 10.1371/journal.pgen.1002198 (PMC3145627; doi:10.1371/journal.pgen.1002198)
Supplement: Table S2 — Summary of variants detected by sequencing in the 256 Sardinians and 120 HapMap Samples. The table summarizes the variants detected by sequencing in different types of biological function. (DOCX) [file pgen.1002198.s005.docx]

|  | **SNP** | | | | | | |  | **Indels** | | | | |  |  |
| --- | --- | --- | --- | --- | --- | --- | --- | --- | --- | --- | --- | --- | --- | --- | --- |
|  | **non-synon** | **synon** | | **3’UTR** | | **5’UTR** | **Other** |  | **3’UTR** | **5’UTR** | | | **Other** |  | **Total** |
| **Sardinian samples** | | | | | | | | | | | | | | | |
| *APOB* | 25 | 17 | | 2 | | 1 | 29 |  | 0 | | 0 | 0 | |  | 74 |
| *APOC1* | 1 | 0 | | 1 | | 0 | 19 |  | 0 | | 0 | 1 | |  | 22 |
| *APOC2* | 1 | 0 | | 2 | | 1 | 31 |  | 1 | | 0 | 1 | |  | 37 |
| *APOE* | 2 | 1 | | 0 | | 0 | 12 |  | 0 | | 0 | 0 | |  | 15 |
| *B3GALT4* | 1 | 1 | | 1 | | 0 | 16 |  | 0 | | 0 | 5 | |  | 24 |
| *B4GALT4* | 1 | 2 | | 10 | | 3 | 25 |  | 0 | | 0 | 3 | |  | 44 |
| *LDLR* | 3 | 9 | | 26 | | 0 | 39 |  | 1 | | 0 | 0 | |  | 78 |
| *PCSK9* | 8 | 5 | | 4 | | 1 | 29 |  | 1 | | 0 | 2 | |  | 50 |
| *SORT1* | 4 | 4 | | 14 | | 0 | 38 |  | 2 | | 0 | 3 | |  | 65 |
| *Overall N (%)* | *46 (11.25%)* | *39 (9.54%)* | | *60 (14.67%)* | | *6 (1.47%)* | *238 (58.19%)* |  | *5 (1.22%)* | | *0 (0%)* | *15 (3.67%)* | |  | *409* |
| *Expected count for 60 individuals* | 25 | 23 | | 37 | | 5 | 150 |  | 4 | | 0 | 15 | |  | 259 |
|  |  |  | |  | |  |  |  |  | |  |  | |  |  |
| **HapMap CEU samples** | | | | | | | | | | | | | | | |
| *APOB* | 22 | 6 | | 0 | | 1 | 24 |  | 0 | | 0 | | 1 |  | 54 |
| *APOC1* | 1 | 0 | | 1 | | 0 | 15 |  | 0 | | 0 | | 1 |  | 18 |
| *APOC2* | 0 | 0 | | 1 | | 1 | 23 |  | 1 | | 0 | | 1 |  | 27 |
| *APOE* | 2 | 0 | | 0 | | 0 | 12 |  | 0 | | 0 | | 0 |  | 14 |
| *B3GALT4* | 1 | 2 | | 1 | | 0 | 15 |  | 0 | | 0 | | 4 |  | 23 |
| *B4GALT4* | 1 | 2 | | 5 | | 4 | 20 |  | 0 | | 0 | | 3 |  | 35 |
| *LDLR* | 5 | 8 | | 22 | | 0 | 33 |  | 1 | | 0 | | 0 |  | 69 |
| *PCSK9* | 5 | 3 | | 5 | | 2 | 31 |  | 2 | | 0 | | 2 |  | 50 |
| *SORT1* | 0 | 4 | | 6 | | 0 | 17 |  | 2 | | 0 | | 3 |  | 32 |
| *Overall N (%)* | *37(11.49%)* | *25 (7.76%)* | | *41 (12.73%)* | | *8 (2.48%)* | *190 (59.01%)* |  | *6 (1.86%)* | | *0 (0%)* | | *15(4.66%)* |  | *322* |
|  |  |  | |  | |  |  |  |  | |  | |  |  |  |
| **HapMap YRI samples** | | | | | | | | | | | | | | | |
| *APOB* | 40 | 20 | | 2 | | 1 | 33 |  | 0 | | 0 | | 2 |  | 98 |
| *APOC1* | 3 | 0 | | 1 | | 0 | 14 |  | 0 | | 0 | | 1 |  | 19 |
| *APOC2* | 3 | 0 | | 2 | | 2 | 48 |  | 1 | | 0 | | 1 |  | 57 |
| *APOE* | 3 | 0 | | 0 | | 1 | 22 |  | 0 | | 0 | | 1 |  | 27 |
| *B3GALT4* | 1 | 1 | | 4 | | 0 | 23 |  | 0 | | 0 | | 5 |  | 34 |
| *B4GALT4* | 2 | 4 | | 4 | | 3 | 31 |  | 0 | | 0 | | 4 |  | 48 |
| *LDLR* | 4 | 8 | | 23 | | 0 | 37 |  | 1 | | 0 | | 0 |  | 73 |
| *PCSK9* | 11 | 9 | | 8 | | 1 | 63 |  | 2 | | 0 | | 3 |  | 97 |
| *SORT1* | 2 | 4 | | 22 | | 0 | 44 |  | 1 | | 0 | | 3 |  | 76 |
| *Overall N (%)* | *69 (13.04%)* | *46 (8.7%)* | | *66 (12.48%)* | | *8 (1.51%)* | *315 (59.55%)* |  | *5( 0.95%)* | | *0 (0%)* | | *20(3.78%)* |  | *529* |
|  |  |  | |  | |  |  |  |  | |  | |  |  |  |
| **All populations** | | | | | | | | | | | | | | | |
| *APOB* | 66 | | 35 | | 4 | 1 | 64 |  | 0 | | 0 | | 3 |  | 173 |
| *APOC1* | 3 | | 0 | | 2 | 0 | 30 |  | 0 | | 0 | | 1 |  | 36 |
| *APOC2* | 4 | | 0 | | 3 | 2 | 59 |  | 0 | | 0 | | 1 |  | 69 |
| *APOE* | 3 | | 1 | | 0 | 1 | 27 |  | 0 | | 0 | | 1 |  | 33 |
| *B3GALT4* | 3 | | 3 | | 4 | 0 | 38 |  | 0 | | 0 | | 2 |  | 50 |
| *B4GALT4* | 2 | | 5 | | 10 | 8 | 45 |  | 0 | | 0 | | 2 |  | 72 |
| *LDLR* | 9 | | 11 | | 31 | 0 | 54 |  | 1 | | 0 | | 0 |  | 106 |
| *PCSK9* | 18 | | 12 | | 13 | 3 | 75 |  | 0 | | 0 | | 3 |  | 124 |
| *SORT1* | 6 | | 4 | | 33 | 0 | 72 |  | 3 | | 0 | | 1 |  | 119 |
| *Overall N (%)* | *114 (14.5%)* | | *71 (9.1%)* | | *100 12.7%)* | *15 (1.9%)* | *464 (59.3%)* |  | *4 (0.5%)* | | *0 (0%)* | | *14 (1.8%)* |  | *782* |
|  |  | |  | |  |  |  |  |  | |  | |  |  |  |
