# Supplementary material for: Fine Mapping of Five Loci Associated with Low-Density Lipoprotein Cholesterol Detects Variants That Double the Explained Heritability
Source: PLoS Genet. 2011 Jul 28;7(7):e1002198. doi: 10.1371/journal.pgen.1002198 (PMC3145627; doi:10.1371/journal.pgen.1002198)
Supplement: Table S3 — Enrichment of rare variants (MAF<0.01). The table lists the number of carriers of coding mutations (MAF<0.01) for each gene in individuals with high or low LDL-C levels. Shaded rows indicate whether a trend for enrichment is observed, although significance was clear only at APOB. (DOCX) [file pgen.1002198.s006.docx]

| **Gene** | **NON-SYN** | | **SYNON** | |
| --- | --- | --- | --- | --- |
|  | High LDL | Low LDL | High LDL | Low LDL |
| *APOB* | 14 | 5 | 16 | 6 |
| *APOC1* | - | - | - | - |
| *APOC2* | 2 | 2 | - | - |
| *APOE* | - | - | - | - |
| *B3GALT4* | 1 | 1 | 1 | - |
| *B4GALT4* | - | - | - | 2 |
| *LDLR* | 3 | - | 4 | 2 |
| *PCSK9* | 4 | - | 1 | 1 |
| *SORT1* | 1 | 3 | - | 3 |
|  |  |  |  |  |
