# Supplementary material for: Fine Mapping of Five Loci Associated with Low-Density Lipoprotein Cholesterol Detects Variants That Double the Explained Heritability
Source: PLoS Genet. 2011 Jul 28;7(7):e1002198. doi: 10.1371/journal.pgen.1002198 (PMC3145627; doi:10.1371/journal.pgen.1002198)
Supplement: Table S4 — Case-control association analysis results. Association signals showing a p-value<0.1 when comparing individuals with high and low LDL values. (DOCX) [file pgen.1002198.s007.docx]

| **GENE_NAME** | **Variant** | **rsID** | **CHR** | **POSITION** | **ALLELES** | **FREQ_ALL1** | **PVALUE** | **FUNCTION** |
| --- | --- | --- | --- | --- | --- | --- | --- | --- |
| *APOE* | APOE-005012 | rs7412 | 19 | 50103919 | T/C | 0.0547 | 0.000132 | NONSYN |
| *APOE* | APOE-001561 | rs769446 | 19 | 50100468 | C/T | 0.0586 | 0.000209 | NON-CODING |
| *APOC1* | APOC1-006930 | rs56131196 | 19 | 50114686 | A/G | 0.1055 | 0.000299 | NON-CODING |
| *APOC1* | APOC1-007030 | rs4420638 | 19 | 50114786 | G/A | 0.1056 | 0.000299 | NON-CODING |
| *APOE* | APOE-004874 | rs429358 | 19 | 50103781 | G/T | 0.0742 | 0.004373 | NONSYN |
| *SORT1* | SORT1-085723 | rs1661278 | 1 | 109658366 | T/G | 0.0117 | 0.008051 | NON-CODING |
| *APOE* | APOE-007325 | rs72654472 | 19 | 50106232 | T/G | 0.0234 | 0.040705 | NON-CODING |
| *APOB* | APOB-026193 | rs72653070 | 2 | 21096258 | A/G | 0.0215 | 0.071213 | SYNON |
| *PCSK9* | PCSK9-002431 | rs11591147 | 1 | 55278235 | T/G | 0.0488 | 0.077582 | NONSYN |
| *SORT1* | SORT1-045464 | rs72646560 | 1 | 109698623 | C/T | 0.0058 | 0.091716 | SYNON |
