# Supplementary material for: Fine Mapping of Five Loci Associated with Low-Density Lipoprotein Cholesterol Detects Variants That Double the Explained Heritability
Source: PLoS Genet. 2011 Jul 28;7(7):e1002198. doi: 10.1371/journal.pgen.1002198 (PMC3145627; doi:10.1371/journal.pgen.1002198)
Supplement: Table S5 — Statistics of detected genotyped and imputed SNPs for each region (+/−250 Kb from gene's transcript). The table summarizes the variants detected and analyzed in each step (sequencing, genotyping, imputation) for each gene. (DOCX) [file pgen.1002198.s008.docx]

|  |  |  | **Sanger Sequence** | **metabochip** | **Sanger + Affy 500K** | | **1000Genomes** | |
| --- | --- | --- | --- | --- | --- | --- | --- | --- |
| ***Gene*** | ***Start*** | ***Stop*** | ***N detected*** | ***N genotyped **** | ***N imputed*** | ***Average r^2*** | ***N imputed*** | ***Average r^2*** |
| *PCSK9* | 55277807 | 55303111 | 124 | 88 | 99 | 0.815 | 446 | 0.784 |
| *SORT1* | 109653714 | 109742086 | 119 | 582 | 81 | 0.851 | 401 | 0.846 |
| *APOB* | 21077805 | 21120450 | 173 | 762 | 79 | 0.880 | 532 | 0.859 |
| *B4GALT4* | 120413278 | 120442442 | 72 | 35 | 90 | 0.861 | 668 | 0.741 |
| *B3GALT4* | 33352894 | 33354580 | 50 | 186 | 66 | 0.959 | 2115 | 0.918 |
| *LDLR* | 11061056 | 11105505 | 106 | 108 | 64 | 0.842 | 556 | 0.816 |
| *APOC1/C2/E* | 50109760 | 50114446 | 138 | 107 | 75 | 0.811 | 348 | 0.744 |
|  |  |  |  |  |  |  |  |  |
| *Total* |  |  | *782* | *1,868* | *554* |  | *5,066* |  |

*Number of successfully genotyped variants
