# Supplementary material for: Fine Mapping of Five Loci Associated with Low-Density Lipoprotein Cholesterol Detects Variants That Double the Explained Heritability
Source: PLoS Genet. 2011 Jul 28;7(7):e1002198. doi: 10.1371/journal.pgen.1002198 (PMC3145627; doi:10.1371/journal.pgen.1002198)
Supplement: Table S6 — Clinical characteristics of study cohorts. The table describes the clinical characteristics for the Sardinians, Finnish and Norwegian populations used for association analyses. (DOCX) [file pgen.1002198.s009.docx]

|  | **SardiNIA** | **Norwegian T2D patients** | **Norwegian unaffected** | **Finnish T2D patients** | **Finnish unaffected** |
| --- | --- | --- | --- | --- | --- |
|  |  |  |  |  |  |
| *N total (% females)* | 5,524 (57.5) | 1,298 (50.4) | 1,495 (49.3) | 1.964 (24.8)^a^ | 6,066 (45.5) |
| *N used for analyses^b^ (% females)* | 5,382 (57.7) | 1,171 (50.7) | 1,436 (49.6) | 1,742 (30.1) | 5,678 (46.3) |
| *age: mean(SD)* | 41.9 (17.3) | 64.3 (12.7) | 63.2 (13.8) | 60.96 (7.9) | 58.54 (8.5) |
| *LDL (mg/dL): mean(SD)* | 124.8 (35.3) | 160.9 (42.9) | 165.0 (43.9) | 128.5 (36.2) | 135.6 (32.7) |
| *HDL (mg/dL): mean(SD)* | 64.3 (14.9) | 49.3 (14.4) | 55.4 (15.7) | 52.2 (15.3) | 59.3 (16.0) |
| *TG (mg/dL): mean (SD)* | 84.6 (51.9) | 195.1 (82.8) | 154.8 (75.0) | 156.5 (90.2) | 115.9 (60.5) |
| *BMI: mean(SD)* | 25.3 (4.6) | 29.1 (4.8) | 26.4 (3.9) | 30.37 (5.4) | 26.93 (4.32) |
|  |  |  |  |  |  |

^a^ Finnish T2D patients sample is skewed towards males because it includes METSIM that enrolled only males

^b^ It excludes samples with missing LDL values or under lipids lowering therapy (except for the Norwegians for which the latest information was unavailable)
