# Supplementary material for: Fine Mapping of Five Loci Associated with Low-Density Lipoprotein Cholesterol Detects Variants That Double the Explained Heritability
Source: PLoS Genet. 2011 Jul 28;7(7):e1002198. doi: 10.1371/journal.pgen.1002198 (PMC3145627; doi:10.1371/journal.pgen.1002198)
Supplement: Table S7 — Association results in the Finnish and Norwegian individuals. The table describes the association results for all SNPs in the diabetics and non-diabetics Finnish and Norwegian samples. (DOCX) [file pgen.1002198.s010.docx]

| **Gene** | **SNP** | **Effect Allele/ Other Allele** | **Freq (SE)** | **Effect (SE)** | **PVALUE** |
| --- | --- | --- | --- | --- | --- |
|  |  |  |  |  |  |
| *PCSK9* | rs11591147 | T/G | 0.039 (0.009) | -0.451 (0.039) | 1.08x10^-30^ |
| *SORT1* | rs583104 | T/G | 0.765 (0.007) | 0.168 (0.017) | 1.20 x10^-23^ |
| *APOB* | rs547235 | A/G | 0.279 (0.013) | -0.045 (0.016) | 0.00525 |
| *LDLR* | rs73015013 | T/C | 0.103 (0.005) | -0.227 (0.234) | 3.05 x10^-22^ |
| *APOE* | rs7412 | T/C | 0.059 (0.013) | -0.596 (0.031) | 8.51 x10^-84^ |
| *LDLR (2^nd^)* | rs72658864 | T/C | 1 | 1 | 1 |
| *APOE (2^nd^)* | rs429358 | T/C | 0.813 (0.006) | -0.151(0.019) | 2.98 x10^-16^ |
| *PCSK9 (2^nd^)* | rs2479415 | C/T | 0.593 (0.032) | 0.048(0.015) | 0.001372 |
|  |  |  |  |  |  |
|  |  |  |  |  |  |
| ***GWAS SNPs*** |  |  |  |  |  |
| *PCSK9* | rs11206510 | T/C | 0.845 (0.008) | 0.115 (0.020) | 5.69 x10^-09^ |
| *SORT1* | rs599839 | A/G | 0.765 (0.007) | 0.168 (0.017) | 1.35 x10^-23^ |
| *APOB* | rs562338 | A/G | 0.187 (0.024) | -0.117 (0.018) | 1.73 x10^-10^ |
| *LDLR* | rs6511720 | T/G | 0.103 (0.005) | -0.224 (0.023) | 1.36 x10^-21^ |
| *APOE* | rs4420638 | A/G | 0.764 (0.015) | -0.175 (0.019) | 1.24 x10^-19^ |
|  |  |  |  |  |  |
