# Supplementary material for: Fine Mapping of Five Loci Associated with Low-Density Lipoprotein Cholesterol Detects Variants That Double the Explained Heritability
Source: PLoS Genet. 2011 Jul 28;7(7):e1002198. doi: 10.1371/journal.pgen.1002198 (PMC3145627; doi:10.1371/journal.pgen.1002198)
Supplement: Table S8 — Loss-of-function and in-frame insertion variants identified by sequencing and their respective frequencies on each population. The table reports the specific loss-of-function and in-frame insertion variants detected in APOB and PCSK9 genes, their genomic position in build36 and frequencies in the 256 Sardinians and 120 CEU and YRI populations. (DOCX) [file pgen.1002198.s011.docx]

| **Gene** | **Variant** | **Position** | **Type** | **Mutated**  **Allele/Normal** | **Freq Sard** | **Freq CEU** | **Freq YRI** |
| --- | --- | --- | --- | --- | --- | --- | --- |
|  |  |  |  |  |  |  |  |
| *APOB* | rs17240441 | 21120280-88 | in-frame insertion | - / TGGCGCTGC | 25% | 23% | 13% |
| *APOB* | chr2:21086604-06 | 21086604-06 | in-frame insertion | - /TGA | -- | 0.8% | - |
| *PCSK9* | rs28362286 | 55301803 | stop-gain | A / C | -- | -- | 0.8% |
| *PCSK9* | rs67608943 | 55284810 | stop-gain | G / C |  |  | 1.6% |
