# Supplementary material for: Fine Mapping of Five Loci Associated with Low-Density Lipoprotein Cholesterol Detects Variants That Double the Explained Heritability
Source: PLoS Genet. 2011 Jul 28;7(7):e1002198. doi: 10.1371/journal.pgen.1002198 (PMC3145627; doi:10.1371/journal.pgen.1002198)
Supplement: Table S9 — Metabochip Genotype Quality Control Details. Statistics of quality controls filters. Note that a marker could have failed more than one check. (DOCX) [file pgen.1002198.s012.docx]

| **Filters** | **#Markers Removed** |
| --- | --- |
| None | 3,277 |
| Call rate <95% | 213 |
| MAF = 0 | 929 |
| HWE pvalue < 10-6 | 0 |
| Excess MI * | 1,262 |
|  |  |
| *Total QCed Markers: 1,868* | |

* excess was defined as >6 Mendelian errors
